# LandScan mosaic enables high-resolution gridded population estimates with explicit uncertainty

**DOI:** 10.1038/s41598-025-28125-z

**Published:** 2025-12-24

**Authors:** Daniel S. Adams, Andrew Zimmer, Joseph Tuccillo, Jessica Moehl, Robert Stewart, Sarah Walters, Budhendra Bhaduri, Peter Li, Marie Urban

**Affiliations:** 1 Geospatial Science and Human Security Division, Oak Ridge National Laboratory, Oak Ridge, TN USA; 2 Department of Earth Sciences, Tennessee Technological University, Cookeville, TN USA

**Keywords:** Environmental sciences, Mathematics and computing, Natural hazards

## Abstract

Gridded population datasets represent high-resolution distributions of human occupancy, enabling informed decision-making across a broad range of fields. These data products are valuable for assessing environmental risk, urban development, disaster preparedness and resource allocation—areas where accurate population estimates directly enhance policy effectiveness and optimize resource distribution. Despite the importance of gridded population datasets, traditional population modeling approaches often overlook inherent uncertainties in the estimation process. This limitation can create a false sense of certainty in population estimates, potentially leading to flawed decisions by those who rely on the data. To address this methodological gap, we introduce a probabilistic machine learning modeling framework, LandScan Mosaic, that explicitly incorporates uncertainty into the population modeling process. Our approach systematically quantifies uncertainty in three key modeling parameters of the LandScan HD gridded population dataset: building use types, floor counts, and occupancy rates. By employing Monte Carlo simulations, we propagate these uncertainties through the modeling process, yielding probability distributions of population counts in place of deterministic point estimates. We demonstrate the practical application of this framework in Iloilo City, Philippines, using structured decision-making techniques and our probabilistic estimates to identify and prioritize areas most affected by projected flooding, supporting targeted interventions that address both economic and social risks. In doing so, we propose a population-specific approach for incorporating confidence into structured decision making processes. Through a comparative analysis with conventional deterministic approaches and point estimate approaches, including LandScan HD and WorldPop, we evaluate how the incorporation of machine learning and uncertainty influences decision rankings. This research advances population distribution modeling by offering a robust, quantitative approach that explicitly accounts for uncertainty in the underlying data, along with guidance for how users can apply uncertainty in their decision-making.

## Introduction

Accurate and high-resolution population data are essential for addressing global challenges related to environmental hazards, political conflict, and growing demand on infrastructure and resources^1^. Gridded population products represent fine-scale distributions of human occupancy which, when combined with other geospatial data products on environmental, climatic, geopolitical, and socioeconomic factors, enable decision-makers to more effectively assess risk, prioritize interventions, and allocate resources^2–6^. The utility of these data extends to historical analyses, near-real-time monitoring and scenario-based forecasting, key tools that support in-depth research and anticipatory planning.

To meet these needs for precise population information, the LandScan High-Definition (LSHD) population distribution dataset developed as an extension of the longstanding LandScan Global product^2,7–9^, produced by Oak Ridge National Laboratory (ORNL). The origins of LSHD trace back to the development of the LandScan USA dataset^10^, which pioneered finer spatial and temporal resolution for population occupancy modeling. Building on this foundation, expanding the approach outside the United States became a natural progression and led to the creation of LSHD. This high-resolution gridded population dataset provides estimates at a 100-meter resolution, offering a level of detail that makes it an invaluable resource for stakeholders who require precise population distributions to guide decision-making processes.

LSHD is modeled through a big data fusion process that leverages both open-source and ORNL produced data to quantify occupancy suitability across built environments and distribute population estimates accordingly^11^. Unlike the broader environmental suitability approach used by LandScan Global, LSHD narrows the definition of habitat to include only areas of built-up surfaces, specifically buildings^11^. This method aims to assess the suitability of individual buildings for human occupancy based on various attributes of the building such as its footprint area, number of floors, and building use (e.g. residential, commercial, industrial, etc.).

**Fig. 1:**
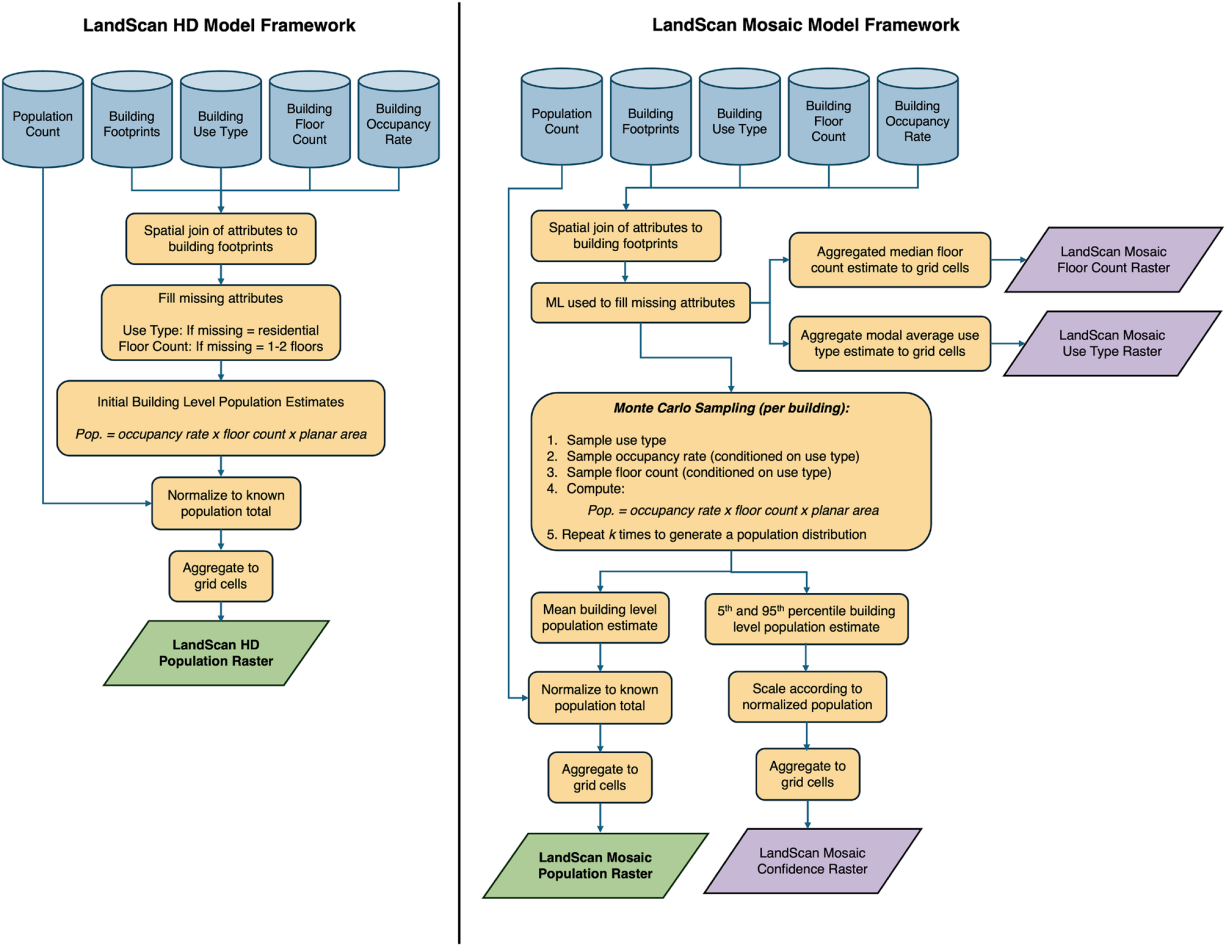
LandScan HD and LandScan mosaic model frameworks.

The modeling approach used for LSHD, illustrated in Fig. 1, has traditionally been deterministic - modelling population as a direct function of occupiable space and density values^11,12^. This approach would be ideal if all input data were complete and accurate, as it would offer transparency and simplicity. However, building attribution is widely recognized as an area with significant data gaps. In many countries, building stock datasets often contain limited information, typically restricted to basic indicators of a building’s presence or absence^13^.

The LSHD model employs two default modeling heuristics to address missing building attribution data^11^. First, when building use type – which informs occupancy rates – is unavailable, the model defaults to classifying such buildings as residential. Second, if floor count data are missing, the LSHD model assumes the building has one to two floors. These assumptions are based on the premise that most buildings in a given country are residential and that residential structures typically have between one to two floors^11^.

While these heuristics are reasonable given the predominance of residential buildings, they are broad assumptions that overlook spatial heterogeneity in building characteristics, thereby introducing uncertainty into the population modeling process. The deterministic nature of LSHD compounds this issue, by producing the same output for identical inputs, limiting its ability to quantify or propagate uncertainty arising from incomplete or imprecise input data. Consequently, decision-makers relying on LSHD receive population estimates that may appear precise but may conceal significant hidden uncertainty due to limitations in the input data. This lack of transparency poses challenges when LSHD is applied to critical contexts where livelihoods and vital infrastructure are at stake.

To address these limitations, we introduce a probabilistic modeling approach that builds upon LSHD and explicitly quantifies and propagates uncertainty throughout the population estimation process, dubbed LandScan Mosaic (LSM). Building on the foundational population estimation function, where population is calculated as the product of occupiable space and occupancy rate density^11,12^, the model for LSM includes a probabilistic framework with Monte Carlo sampling. Rather than assigning fixed values, the model probabilistically determines key building attributes by drawing from likelihood distributions produced by machine learning models developed in this work, while occupancy rates are sampled from statistical distributions derived from ORNL’s Population Density Tables (PDT) project^14^. By performing multiple iterations of this simulation, the model generates a range of plausible population estimates rather than a single deterministic value, providing a more robust representation of uncertainty in population distribution. To demonstrate the practical utility of this framework, we apply it to a real-world scenario, examining and ranking geographic regions based on populations susceptible to flooding and comparing the results with those derived from traditional point estimate population datasets.

## Literature review

The purpose of this literature review is to contextualize our approach within other existing population modeling efforts and supporting research. First, we compare global gridded population models, including the Global Human Settlement Population Grid (GHS-POP)^15^, High-Resolution Settlement Layer (HRSL)^16^, WorldPop (WP)^17^, and LandScan^7–9^, and detail their methodologies. Among these approaches, we identify a common tendency to overlook uncertainty quantification for data inputs (e.g. settled areas, land-use/land cover, building form, function, and occupancy) that limits the ability to evaluate confidence in derived population estimates. We then propose ways in which uncertainty can be propagated through the LSM model through its data inputs, specifically building attribution and occupancy estimation. One key focal area is the use of machine learning to infer building use type and floor count from remote sensing and geospatial data. These attributes are essential for accurate population modeling using the LSHD modeling framework^11,12^ but are often incomplete or missing at scale^13^. Another focal area is expanding usage of the occupancy rate ($\text {people}/1000\text {ft}^2$) distributions derived from PDT. These empirically derived beta distributions provide a probabilistic basis for estimating population densities across different building types, serving as an essential component of our revised modeling framework. This review establishes the foundation for a probabilistic approach to high-resolution population estimation, which integrates machine learned building attributes and PDT-derived occupancy rates within a Monte Carlo simulation framework.

### Population modeling efforts

Historically, gridded population modeling has focused on the spatial disaggregation of administrative population totals from national census or population survey data^6,17,18^. Advancements over the past decades have largely focused on refining suitability weighting for identifying areas inhabitable by people. This process broadly involves the identification and quantification of factors that influence human habitation patterns, such as settlement area, urban/rural extents, land-use and land cover, and building function. In this section, we review the state-of-the-art global gridded population datasets and their methodological advancements^18^. The first set of of models primarily rely on representations of built-up areas and building density to disaggregate administrative population counts.

The Global Human Settlement Population (GHS-POP) dataset is an open-access global population model that was among the first to produce high-spatial resolution (100m) multi-temporal estimates. Unlike traditional gridded population datasets that rely solely on administrative boundaries and census data, GHS-POP integrates satellite-derived built-up area data from the Global Human Settlement Layer (GHSL) to enhance the spatial dissaggregation of population^15,18^. The GHS-POP framework employs a three-tiered disaggregation approach, prioritizing population allocation to built-up areas when available. In regions lacking built-up data, population is distributed either through areal weighting or assigned to the geographic centers of small administrative units.

In relation to our work, GHS-POP operates as a deterministic model that assumes uniform population densities across built-up areas, weighting spatial units solely based on concentrations of built-up surfaces. It does not account for variations in building characteristics – such as use type, floor count, or occupancy rates – which are critical for capturing spatial heterogeneity in population distribution within and among human settlements. Moreover, GHS-POP lacks any formal mechanisms for quantifying uncertainty in its population estimates.

Similar to GHS-POP, Meta provides HRSL at a 30m resolution for 160 countries around the globe. The HRSL modeling approach mimics that of GHS-POP by disaggregating census data to human settlements but with the added criterion of the density of detected buildings within each grid cell^16^. The model benefits from a highly accurate building detection approach, resulting in a reliable gridded population data product that represents an improvement over GHS-POP’s dissagregation strategy.

In the context of our own work, HRSL contains similar limitations to that of GHS-POP in that buildings are all treated equally and the only weight derived is based purely on building density. In its current state, HRSL lacks a mechanism for reporting uncertainty, though this has been identified as a direction for future development.

Dasymetric gridded population models reallocate census data spatially using weighting layers derived from ancillary data sources. In contrast to GHS-POP and HRSL, which rely solely on built-up area information, dasymetric models account for the idea that some areas may be “more inhabitable” than others based on landscape features including land use/land cover, night-time lights, road networks, and climate characteristics.

A key example of the dasymetric modeling approach is WP, whose global product employs a Random Forest-based dasymetric model that distributes census-based population counts at approximately 100m and 1km resolutions using a variety of remotely sensed landscape features^17,18^. Despite its methodological advancements, WP’s global model output remains deterministic, lacking a formal framework for uncertainty quantification. However, to explicitly model uncertainty in select regions of the world, WP has developed a Bayesian hierarchical approach that integrates household survey data and building footprints to estimate population distributions with credible intervals^19^. While this method improves accuracy, its reliance on household survey data limits its scalability for global application, given the inconsistent quality, coverage and availability across countries^20,21^.

LandScan Global (LSG), developed by Oak Ridge National Laboratory, has historically used a proprietary, expert-driven dasymetric approach, where population weights were derived via expert elicitation of suitability for human habitation among landscape features^7–9^. Recent methodological updates have transitioned LandScan toward a machine learning-driven disaggregation framework^22^. The latest LSG approach employs Random Forest^23^ and XGBoost^24^ models, trained and tested at 30 arc-second resolution (approximately 100m). Unlike WP, which trains its Random Forest models at administrative unit levels before applying them at finer resolutions^17^, LSG trains directly on previous years’ LSG outputs, using the preceding population estimates to predict future distributions. This strategy effectively encodes and refines historical expert-driven patterns, thereby ensuring temporal consistency across annual products. While the LSG methodology enhances spatial consistency across years, it remains fundamentally deterministic, offering no current mechanism for quantifying the uncertainty associated with either the original expert judgments or the current machine learning predictions.

The LSHD gridded population model is distinct from built-up area (GHS-POP, HRSL) and dasymetric modeling (WP, LSG) approaches in that it estimates suitability for human habitation at the granular level of building footprints with respect to their form, function, and occupancy. The ease of aggregating building-level population estimates to areal units in turn enhances spatial resolution of the final gridded population product (3 arcseconds, or roughly 90m)^25^. As described by Tuccillo et al. and Urban et al.^11,12^, the weights used for disaggregating population counts across buildings are derived from a simple product of three key factors: building footprint area, building floor count, and building use type occupancy rate ($\text {people}/1000\text {ft}^2$) via the PDT project^14,26^. Once population values are allocated at the building level, they are aggregated into sum values to generate the final LSHD gridded raster. However, as currently implemented, LSHD lacks a formal mechanism for quantifying uncertainty or propagating it to population estimates.

A common limitation across all surveyed models (GHS-POP, HRSL, WP, LSG, LSHD) is the presence of epistemic, or model-based, uncertainty in the population estimates. Epistemic uncertainty refers to a lack of complete knowledge about data used within a model^27^, in this case a consequence of how built-up areas and ancillary landscape features are characterized. Central to this problem are modeling choices like reliance on hard labels (e.g. one use type per building or pixel) and default values in cases of missingness (e.g. a fallback label of “Residential” for unlabeled buildings), which ignore the model’s uncertainty about these assignments and overlook the full range of plausible characterizations. Additionally, aleatoric uncertainty, or inherent uncertainty in feature attributes^27^ such as the occupancy rates used in LSHD, goes unaccounted for in final population estimates.

Addressing the limitation of unquantified uncertainty is the primary focus of the LSM model. Rather than relying on single point estimates for building attributes, LSM explicitly represents uncertainty across key inputs to the population model. For building characteristics like use type and floor count, LSM propagates epistemic uncertainty through the probability distributions that reflect model uncertainty in classification. For occupancy rates, LSM incorporates aleatoric uncertainty that captures the inherent variability in occupancy across buildings of the same type. By sampling from these distributions, LSM produces an ensemble of population estimates that collectively characterize both epistemic and aleatoric uncertainty associated with population estimation.

### Building level attribution and occupancy estimation

Building use type, floor count, and occupancy form the spatial basis for allocating population to built-up areas in the LSHD and LSM models. Floor count, when combined with building footprint area, provides a volumetric estimate of the floor area of each building. Occupancy estimates of people per 1000 sqft, mapped to buildings by use type, are then used to assign population weights (proportion of total population) at each building location. Due to unobserved, missing, or incomplete data throughout the world, each of these attributes must often be predicted using machine learning (ML) models^14,28^. ML predictions take various forms, from label probabilities (building use type and floors) to parameter estimates of statistical distributions (occupancy). Each of these prediction types in turn has its own form of variability that can be leveraged to refine population estimates in the LSM model.

Building footprints are commonly available as a two-dimensional planar representation of a building’s extent, and as such, they are not accompanied by estimates of height or number of floors. Compounding this challenge, building use types are often available through a patchwork of data sources like OpenStreetMap (OSM)^29^ that do not offer complete coverage of building footprints^11^. Significant advancements in built environment characterization in the fields of remote sensing and urban morphometrics offer solutions to these problems^28,30–39^. Much of this progress has been driven by tree-based machine learning models, which have dominated the literature for inferring both a building’s intended use and height characteristics^28,30–33,35,36,38–44^. These developments have substantially improved the accuracy of building attribution across various spatial scales. However, many of these approaches rely on features that, while highly predictive, are not globally available^30,35,42–44^. To address this limitation, we review the most relevant advancements in building attribution that leverage globally scalable features derived from building footprints, with the goal of enhancing global population modeling.

Several notable studies and data production efforts have focused on characterizing building stock and its attributes using features collectively referred to as building morphology^33,34,36–39^. Building morphology encompasses a set of descriptors that define the physical appearance of a building’s footprint. These features range from basic geometric characteristics, such as: footprint area, perimeter, and vertex count and contextual details: the average size of surrounding buildings. Additionally, spatial descriptive statistics such as: the distance between buildings, the number of buildings within certain buffer distances, and general patterns of building placement and density, further enhance building morphology analysis^45^.

One prominent example is the USA Structures dataset^33^, which leverages building morphology to impute missing building use type attributes absent from parcel data. Similarly, studies by Adams et al.^38,39^ demonstrated that intrinsic building morphology characteristics, combined with labeled data from open-source repositories like OSM^29^, enable highly accurate residential and non-residential classifications across Ethiopia, Lebanon, Jordan, Türkiye, the Philippines, and Malaysia. Furthermore, research by Stipek et al.^28,46^ found that intrinsic morphological features can be used not only to estimate building height but also to classify buildings into broad descriptive categories, such as ”tall” or ”short.”

In the context of our work, there is strong empirical evidence supporting the use of building morphology features for estimating both building height and use type characteristics. Although the reviewed studies do not explicitly address uncertainty quantification, the tree based methodologies they employ (including Random Forest classifiers and XGBoost regressors/classifiers) can readily be adapted to output probability values rather than point estimates. This capability enables the integration of uncertainty quantification into the LSM modeling framework, thereby enhancing the robustness and interpretability of population estimates.

Occupancy rates (e.g. $\text {people}/1000\text {ft}^2$) complement building attributes such as use type and number of floors, offering insight into the expected number of occupants within a structure at a given time. While these rates have seen widespread consumer-facing applications - such as Google’s ‘Popular Times’ feature - they remain largely underutilized in major gridded population datasets including LandScan Global, WP, and GHS-POP^7,8,15,17^. In contrast, LSHD and LSM each explicitly incorporate occupancy rates as a key model parameter.

Patterns of human occupancy are influenced by a building’s intended use—whether residential, commercial, or otherwise—a function of its spatial location within the built environment^37^. For instance, residential buildings typically have higher occupancy during evenings and weekends, aligning with non-working hours^47^, whereas commercial buildings typically more populated during standard business hours^48^.

To account for this variability in occupancy rates, the Population Density Tables (PDT) project provides probabilistic estimates of ambient human activity (e.g. $\text {people}/1000\text {ft}^2$) at scale across 8 land-use classes and 65 facility-types^12^.

Built on a Bayesian statistical framework^14^, PDT relies on observation models built from facility-specific data to provide robust information about how humans occupy space. The PDT facility-specific observational method accommodates building specific, neighborhood, city, administrative, national or regional “snapshots” of ambient occupancy rate^14,26^. Through open authoritative sources such as country statistical census or surveys^49^, non-governmental surveys and reports^50^, news articles, journal articles, ethnographies, real estate databases, websites for commercial companies and educational research and anecdotal sources such as social media, as well as novel applications of computer vision and geospatial feature extraction^51–53^, PDT applies a diversity of inputs to inform its systematic observation workflow^26^. These observations are harmonized and serve as inputs to a Bayesian ML model that updates the parameters of statistical (beta) distributions for estimating occupancy rates (people per 1000 sqft) by country and facility type. Further discussion of the PDT Bayesian framework to establish and handle observation updates of the prior is found in the “Methods” section.

Applied within the context of LSM, PDT provides a comprehensive set of empirically derived occupancy rate distributions, which are integrated with building stock datasets. This process effectively spatializes the occupancy rates to create a gridded population distribution capable of reflecting occupancy shifts across both time and space. While the LSHD model relies on single PDT point estimates (the 50th percentile of the distribution), LSM incorporates the full range of occupancy rates for a country/facility type by sampling values from their representative distributions. This approach is particularly well-suited for supporting uncertainty quantification in population estimation and – in combination with variability in building height and use predictions – enables the propagation of that uncertainty throughout the LSM model.

### Applications of uncertainty in decision-making

The operational role of uncertainty in population distribution data is underdeveloped. Gridded population datasets differ in their modeling strategies, input data sources, and calibration targets. Even official census data, the gold standard in population counts, are subject to sampling error, enumeration bias, and a temporal lag. When uncertainty is quantified, it is reported as either metadata or confidence intervals rather than being incorporated into analytical workflows or decision rules. As a result, users are left with population estimates that implicitly assume equal confidence everywhere, even when this is demonstrably untrue.

One of the few examples of operational use is Leasure et al.^54^, who proposed using upper and lower bounds of population estimates to define ”high” and ”low” allocation scenarios for vaccine delivery. While effective for capturing the extremes and suitable for reducing the risk of vaccination shortages, a binary framework like this only presents a switch-like uncertainty implementation for decision-makers rather than a gradient. It would not be able to express whether the underlying estimates systematically overcount or undercount settlements, nor would it provide a mechanism for weighting the consequences of misestimation differently across contexts or spatial regions. Many decision-making scenarios require continuous risk-responsive adjustments rather than extreme all-or-nothing scenario switches. At present, no established guidance exists for how uncertainty in population estimates should be integrated into structured decision making (SDM) frameworks where trade-offs must be communicated, justified, and reproducible.

When formulating guidance for how to effectively leverage quantified uncertainty in population data products, one must consider the particular consequences of error in population estimation. We assert that in many operational contexts, undercounting and overcounting population do not carry equal weight. Undercounting exposed populations in flood evacuation planning may result in insufficient capacity and preventable casualties, whereas overcounting primarily wastes resources. The reverse may be true in other contexts, where overestimation in political apportionment could yield overrepresentation and inequitable resource allocation. What these scenarios share is directional asymmetry: one type of error systematically matters more than the other.

To address this gap, we look to how other fields formalize decision-making under asymmetric risk and uncertain conditions. Post-modern portfolio theory formalizes the idea that losses carry greater consequence than equivalent gains, motivating downside-focused metrics such as the Sortino ratio^55^ and Conditional Value-at-Risk^56^. This rationale mirrors that of our population estimation context, where undercounting (e.g. for flood evacuation or mitigation planning) may carry more real world impact on decision-making than overcounting, or vice-versa depending on differing operational objectives. Guidance for users of quantified uncertainty in population datasets should enable decision-makers to emphasize one direction of error over the other, to better align with their objectives.

Confidence derived weighting frameworks in finance, such as the Black-Litterman model^57^ and the Meucci’s Fully Flexible Views^58^, demonstrate how baseline estimates can be systematically adjusted based on both evidence strength and decision-maker confidence. Finally, convex risk response frameworks, such as those discussed in Taleb’s dynamic hedging work^59^, show that decision systems may rationally increase or decrease sensitivity to uncertainty depending on the direction and magnitude of skew. These ideas are directly relevant to population estimates with non-Gaussian and asymmetric percentile spreads.

When reviewing such sources holistically, we assert that uncertainty should not be treated as a passive annotation or binary scenario, but instead as a structured, directional, and context-dependent contributor to decision making akin to how it is used in financial management. Motivated by these examples from precedents in finance, we introduce a continuous, uncertainty-weighted adjustment in Eq. (29), that allows users to tune population estimates according to the (1) confidence in the estimate, (2) the directional asymmetry in uncertainty, and (3) the operational costs of undercounting versus overcounting. Thus, filling a gap in current population modeling practice by enabling explicit alignment between uncertainty structure and decision objectives. For example, in flood mitigation and evacuation planning, uncertainty in the number of people in flood-prone areas can have asymmetric consequences: undercounting may lead to insuifficient transport capacity or shelter space, while overcounting may result in unnecessary resource allocation. This uncertainty-weighted adjustment enables explicit alignment between population uncertainty and decision objectives.

## Results

In this section, we present the outcomes of the demonstration, including the performance of the building use type and floor count models, as well as the final gridded population dataset results. The results for the use type and floor count models can be measured quantitatively using validation holdout sets, whereas the gridded population datasets lack any formal validation as building level population survey data rarely exist. We qualitatively assess the population distribution by comparing against satellite imagery to verify that these align and correspond with the built environment. Beyond visual comparison, we also evaluate whether the rules conform to typical intra-urban dynamics by comparing with population concentrations and distributions based on expected land use patterns and infrastructure. Additionally, where available, we check street-view imagery to verify floor count and use-type accuracy. This type of checking remains essential in data-sparse environments without ground-truth data to validate with. Finally, we detail the results of the structured decision making analysis, intended to highlight the practical real world implications the revised modeling approach may have.

### Floor count model results

**Fig. 2:**
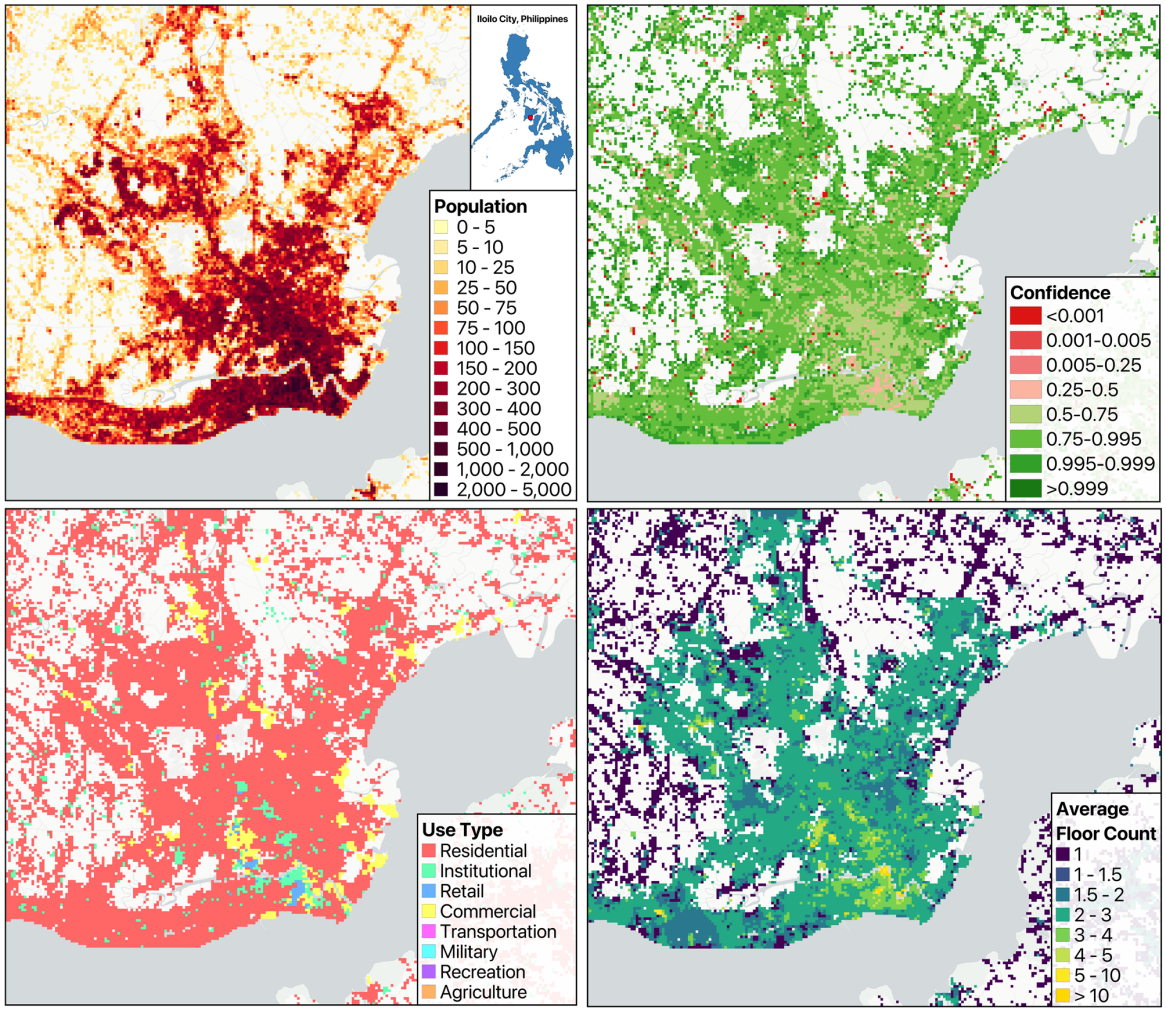
LandScan mosaic model outputs for Iiolio City, Philippines. Top Left: uncertainty adjusted population estimates. Top Right: Confidence raster, expressed as the inverse spread between the population estimates 95th and 5th percentiles, where values close to 1 are higher confidence and values close to 0 are low confidence. Bottom Left: Area Weighted Modal Average of Use Type of Buildings. Bottom Right: Building Area Weighted Average Floor Count.

Table 1 presents the results of the floor count model, evaluated using a 20% randomly selected holdout set with hyperparameters selected via GridSearchCV. For comparison, we also include results based on the two commonly used heuristics for LSHD, which assign all buildings either 1.5 or 2 floors. These heuristic values were selected subjectively in the past, based on the most commonly observed floor counts for residential structures^11^. As evidenced by the MAE and RMSE metrics, the floor count model outperforms both of these LSHD heuristics, demonstrating a clear improvement over the original LSHD floor count approach. In Fig. 2 we present the average predicted floor counts per 100m cell, weighted by building area in Iloilo City, Philippines.

**Table 1:** Floor count results (CV model/LSHD Heuristic 1.5/LSHD Heuristic 2) in the Philippines.

RMSE	MAE
2.04/3.33/3.24	0.63/1.14/1.16

### Use type model results

We provide the results of the classification use type model in Table 2, reflecting performance on a 20% randomly selected holdout set with hyperparameters chosen via GridSearchCV. In the table we also provide the would-be results from the LSHD heuristic for labeling all buildings as simply residential to give an idea of the general model parameter input difference between the two LSHD modeling approaches. The classification model exceeds the classification results across all classes. Naturally, by labeling everything residential, all other classes receive functional F1 scores of 0.0. As such, with our simple A/B comparison being between the modeling approach of LSHD and the revised LSM approach, success is simply defined as achieving similar levels of performance in the residential category for the classification model as calling everything residential while anything above 0.0 in all other categories is an improvement. Given that the classification model not only achieves better performance in the residential category but also maintains F1 scores above 0.5 across all categories, it can be regarded as a substantial improvement over the LSHD approach. In Fig. 2 we present the most frequent predicted type of building per 100m cell, weighted by building area in Iloilo City, Philippines.

**Table 2:** Use type classification results (Random Forest/LSHD Heuristic) in the Philippines.

Class	Precision	Recall	F1-score
Residential	0.98/0.89	0.97/1.00	0.98/0.94
Institutional	0.75/0.00	0.77/0.00	0.76/0.00
Retail	0.72/0.00	0.61/0.00	0.66/0.00
Commercial	0.79/0.00	0.92/0.00	0.85/0.00
Transportation	0.57/0.00	0.66/0.00	0.61/0.00
Military	0.89/0.00	0.85/0.00	0.87/0.00
Recreation	0.76/0.00	0.45/0.00	0.56/0.00
Agriculture	0.78/0.00	0.69/0.00	0.73/0.00

### Gridded population model output

**Fig. 3:**
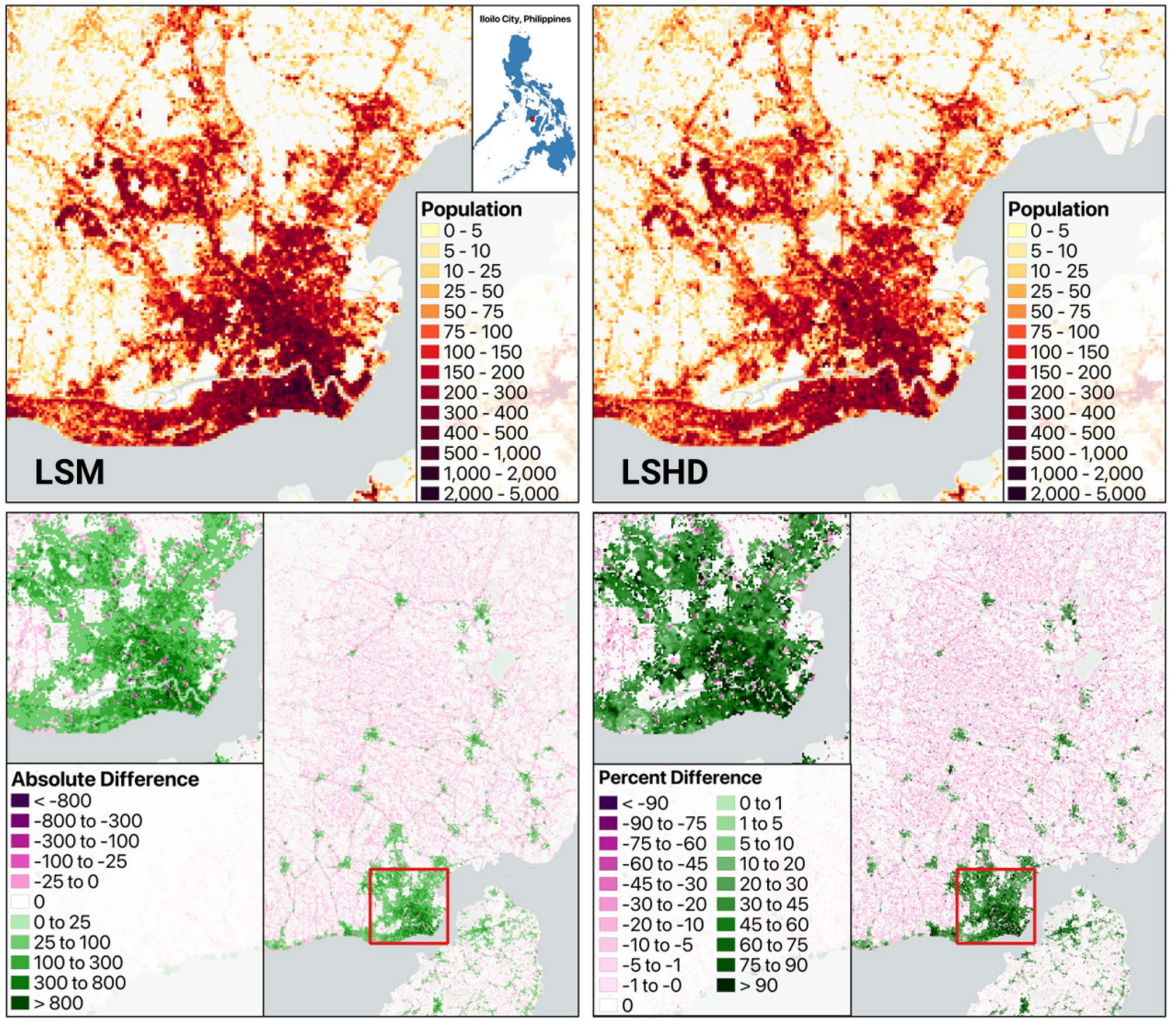
Differences between LSM and LSHD population distributions.

Figure 2 presents the gridded LSM population model output for Iiolio City, Philippines, averaged over ten thousand simulated estimates. The percent change map in Fig. 3 highlights areas where the LSM model redistributes population, shifting density away from the sparsely populated rural villages towards more urbanized areas with more infrastructure.

The population distributions produced by both models are broadly consistent, an expected outcome given that both approaches draw from the same aggregate population data. However, key differences emerge at finer spatial scales. The LSM model enables more realistic allocation of population at the building level through the inclusion of use-type and floor count data. This data-driven refinement leads to localized improvements that better reflect observable patterns in the built environment and known urban structure. Qualitatively, the LSM output appears to represent human occupation across space more accurately. It more closely aligns with high-resolution satellite imagery and conforms to both intra- and inter-urban patterns, including higher density near commercial areas and key infrastructure. Notably, at the inter-urban scale, LSM corrects for unrealistic uniformity in the LSHD model by reallocating populations from smaller town centers to larger cities, which can sustain a higher population density due to vertical development and denser infrastructure^60^. The improvements in floor count and use type modeling, as reported in Tables 1 and 2, provide further confidence that these microscale adjustments result in more accurate population data products.

These enhancements directly impact applied decision making. In scenarios such as disaster response, infrastructure planning, or public health resource allocation, a lack of uncertainty quantification can propagate into decision-making. By addressing uncertainty and improving the fidelity of population estimates at the building level, the LSM approach offers a more robust and actionable dataset for various applications.

### Population model confidence index output

Figure 2 shows the confidence levels in the gridded population model outputs for Iiolio City, Philippines, normalized between 0 and 1, where 0 indicates the lowest confidence and 1 is the highest. Confidence is computed as the inverse spread of the 95th and 5th percentiles of the LSM prediction distribution. Since the LSHD model does not provide uncertainty quantification, these confidence estimates are available only for LSM.

Confidence values across Iiolio city vary, with a notable area of lower confidence in the southern region, known as ’Iiolio City Proper’. This area is densely populated and characterized by diverse building use types and floor counts, reflecting the complexity typical of urban cores in rapidly developing cities. In contrast, higher confidence is observed in areas further from the city center, where the built environment and building characteristics are more homogeneous and predominantly residential.

These spatial patterns are common across many urban areas, where centres of cities tend to be densely populated and functionally diverse, resulting in more modeling uncertainty. Confidence estimates such as these can help guide downstream applications of population data by informing how uncertainty can be applied in decision-making processes.

**Fig. 4:**
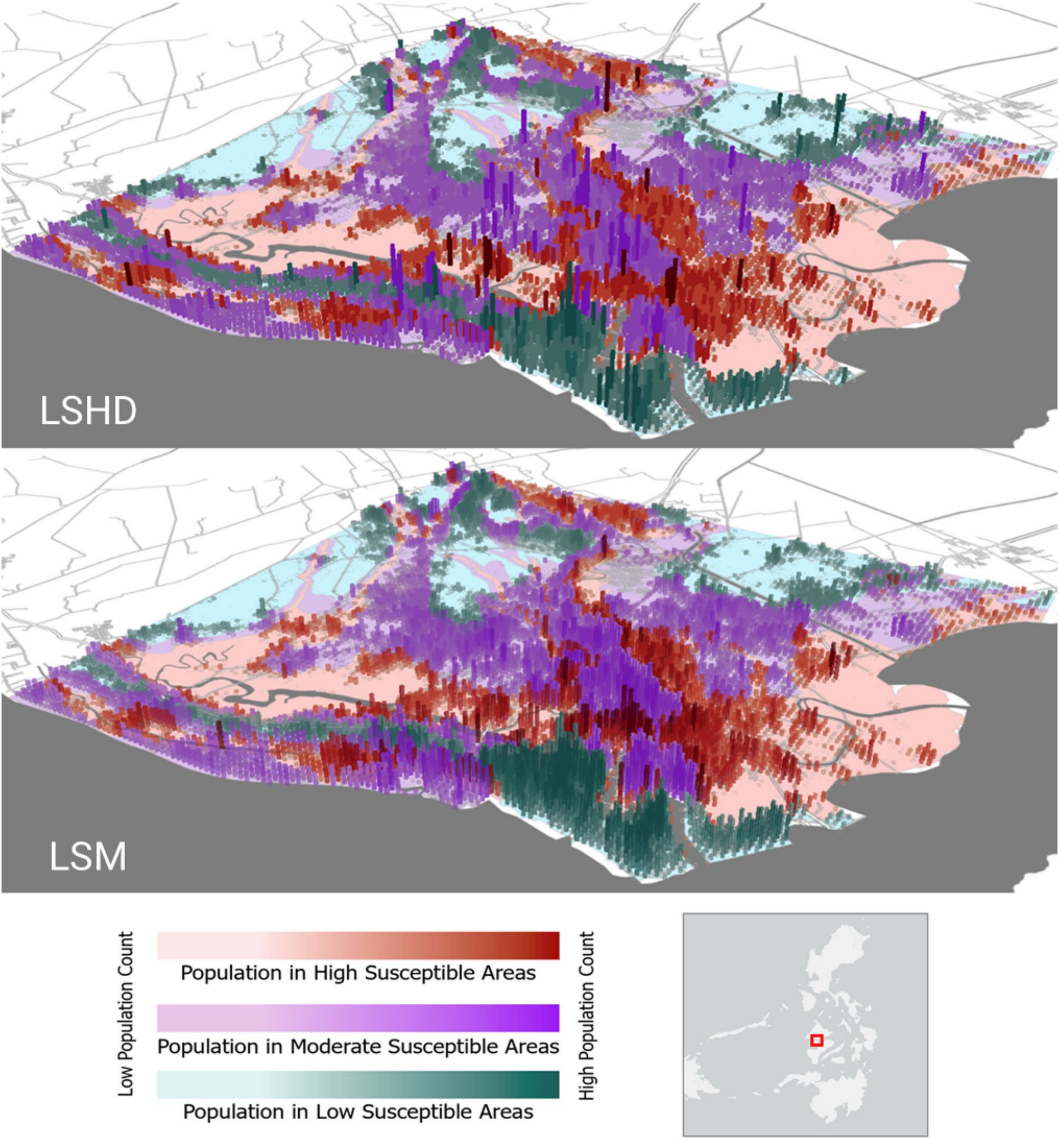
Comparison of gridded population outputs across flood susceptibility zones.

**Fig. 5:**
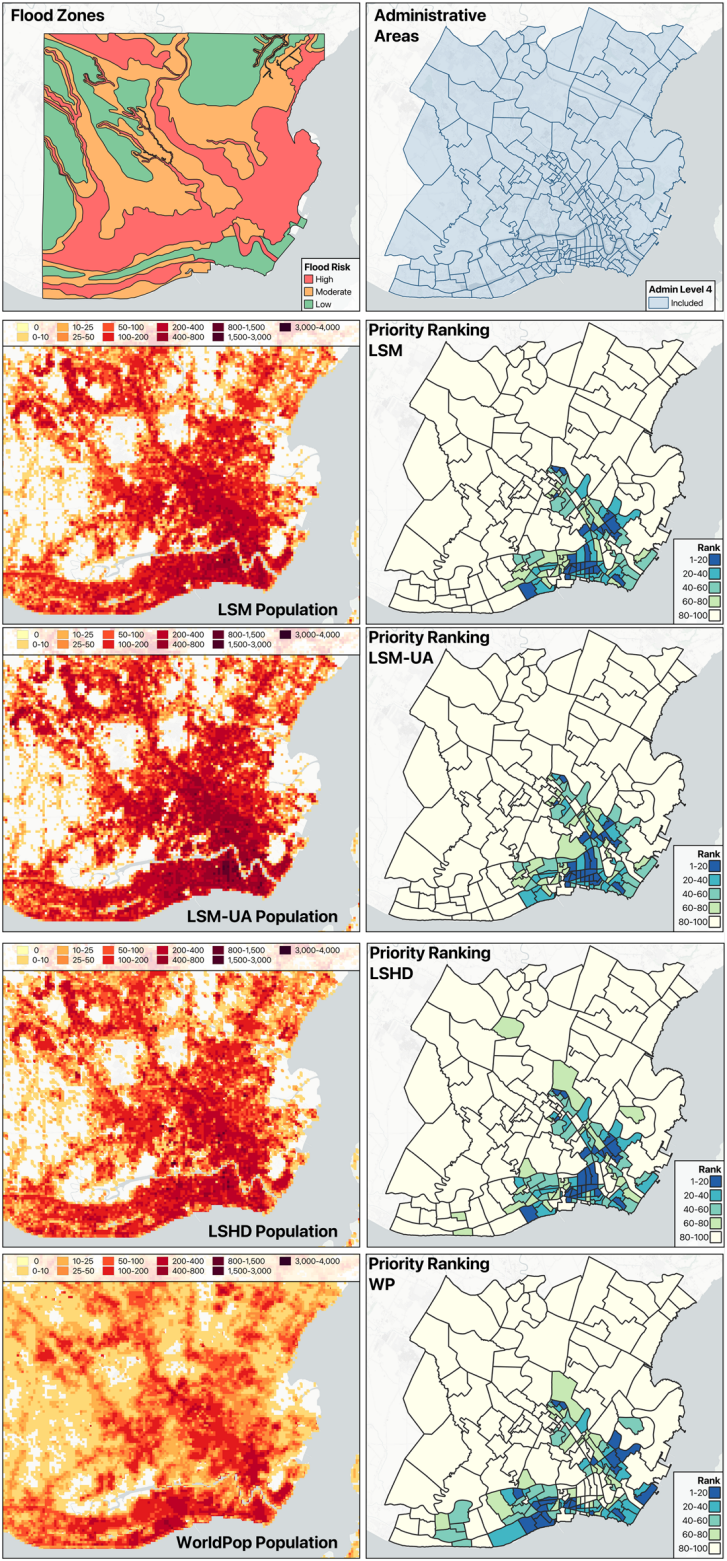
Structured decision-making using flood risk, LSM, LSM-UA, LSHD and WP population data.

### Structured decision making output

Table 3 presents the estimated number and proportion of people exposed to flooding across susceptibility categories, as derived from the LSHD, LSM and WP datasets. While the absolute population counts differ between the two LandScan products, a distinction already illustrated in our comparison figures, inclusion of the WP dataset provides an external reference indicating that relative distributions across flood risk zones remain stable across the different methodologies.

Figure  4 shows that LSHD exhibits sharp spikes in population density, with abrupt declines in neighboring pixels, while the LSM output smooths these transitions, maintaining peak values but tapering off more gradually. Although initial comparisons suggested substantial differences in population allocation per zone, aggregating exposure by flood susceptibility category reveals that both datasets assign comparable proportions of their total population to high, moderate, and low-risk areas. This reflects the fact that, despite LSM concentrating more population in urban areas, the overall exposure distribution remains broadly consistent at the city scale.

Figure 5 shows that the choice of population dataset has an observable effect on the final spatial prioritizations within the municipality. The LSM and its uncertainty-adjusted version (LSM-UA), generally show strong agreement, where the resulting highest priority administrative areas are concentrated in the urban core of Iloilo City. Incorporating uncertainty through the LSM-UA adjustment slightly increases priority ranks for several central administrative areas and decreases at the periphery, reflecting the higher likelihood that populations in dense urban zones exceed the median estimates reported in LSM. In contrast, ranking based on the LSHD dataset mirror the dataset’s underlying population spikes, which arise from how building information are represented. Where building data are available, populations are fully modeled. Whereas when unavailable default heuristics have an observable effect, creating abrupt transitions that overemphasize certain areas within the municipality. Consequently, LSHD prioritizes several administrative units outside the urban core of the municipality.

**Fig. 6:**
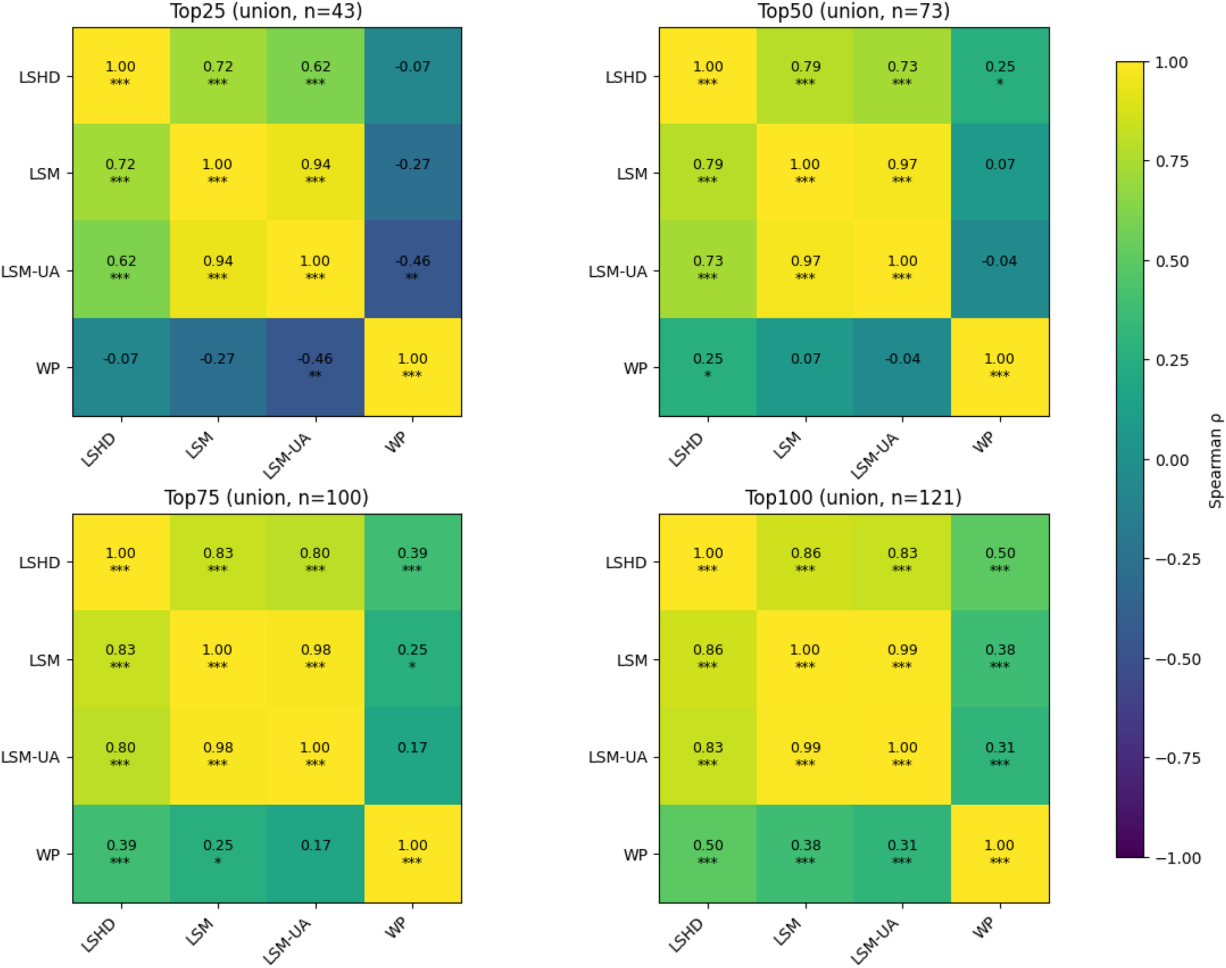
Spearman rank coefficient across administrative units prioritized. Asterisks indicate statistical significance ($${*}^{p}\,<$$ 0.05; $${**}^{p}\,<$$ 0.01; $${***}^{p}\,<$$ 0.001).

When comparing these results with those derived from the WP dataset, we observe broader and generally higher population estimates across the municipality. This divergence likely reflects fundamental differences in what each dataset represents and how it is modeled^11^. WP applies a top-down dissaggregation of census totals using spatial covariates^17^, producing a static snapshot of residential population distribution. In contrast, the LandScan framework models ambient population, representing an average of day-time and night-time estimates derived from building-level information. As a result, WP yields smoother, more generalized spatial patterns, while the LandScan products capture finer building-level variation associated with human activity. These differences manifest in the priority rankings, as seen in Fig. 5. This is further reinforced in Fig. 6, where the Spearman’s rank correlation coefficients between the priority rankings derived from each population dataset across the top 25, 50, 75, and 100 administrative units. Each panel is based on the union of administrative units appearing within the top *N* of any dataset. High correlations are observed between LSM and LSM-UA, reflecting strong consistency in ranking even when uncertainty adjustments are applied across all *N* levels. Moderate to High correlations between LSHD and LSM indicate that while broad prioritization patterns remain aligned, reordering occurs among the top-ranked administrative units. Correlations involving WP are low to near zero. This indicates that despite observing city-scale similarities in population magnitude within each flood risk zone, the local patterns differ significantly from those of the LandScan products. While agreement improves as more units are considered, it is the top-ranked areas that would guide resource allocation decisions, precisely where the divergence between datasets is most pronounced. These results demonstrate that the introduction of the LSM population modeling approach and its subsequent outputs, will have notable implications for users of population data in applications where localized prioritization and exposure assessments are critical.

**Table 3:** Total population exposure by flood susceptibility zone for WP, LSM and LSHD estimates.

Susceptibility	WP	WP %	LSM	LSM %	LSHD	LSHD %
High	186,398	29.97%	317,204	29.97%	269,338	29.46%
Moderate	248,914	43.61%	481,986	45.55%	424,241	46.41%
Low	135,513	23.74%	259,055	24.48%	220,586	24.13%

## Discussion

In this demonstration, we have shown that the LSM framework provides a measurable improvement in representing ambient population distributions relative to LSHD. By leveraging the class probabilities from our building attribution models and repeated sampling from the PDT occupancy rates, LSM produces spatial distributions that better represent the spatial heterogeneity of expected human settlement patterns. Extending this capability, we introduced guidance for decision makers using LSM to incorporate the magnitude and directional nature of modeled uncertainty into their decision-making workflows. Together, these advancements provide both an improved baseline representation of population exposure and a flexible framework for tailoring that exposure to specific risk positioning and priorities of differing decision contexts.

Future work should explore how different formulations of population uncertainty influence structured decision-making outcomes. Sensitivity analyses of the uncertainty adjustment parameters used and their interactions with varying hazard intensities or spatial decision thresholds could reveal to decision makers how uncertainty propagates through prioritization frameworks.

A notable finding from the comparative prioritization results is the divergence observed between WP and LandScan data products. While the present study does not seek to determine the underlying causes of these differences, one plausble explanation is the contrast in modeling foundations, where the WorldPop group uses a suite of ancillary geospatial covariates to guide their suitability weightings, whereas the LandScan methods presented are building-only frameworks. These modeling priorities may emphasize different aspects of human settlement patterns, leading to the observed shifts in prioritization observed in our demonstration. Further investigation of these types of divergences could guide efforts of future work.

While our revised framework represents a significant advancement in the precision and accuracy of population distribution modeling, several areas remain readily available for improvement. First, the probability calibration for the use type and floor count models remains an open challenge. Currently, our random forest models produce probability distributions based on country-specific training data obtained from a variety of sources; however, we lack external validation such as municipal records or comprehensive census data to assess whether these distributions are truly representative of ground truth conditions. Furthermore, our approach for deriving floor count probabilities, treating it as a regression problem, was chosen to overcome missing floor labels that inhibit a strict classification setup. While this approach enables the model to learn from more available data, it also means that the predicted distributions should be interpreted as relative confidence measures rather than well-calibrated probabilities in the traditional sense. Future work will focus on incorporating probability calibration techniques and identify reliable external datasets to validate and improve the interpretability and robustness of these probabilistic outputs.

Additionally, while we model the probabilities of use type and floor count independently, we explicitly compute the joint probability of these variables to account for inherent relationships between these two variables. This enables us to derive the conditional probability of a building having a specific number of floors given its use type - a necessary step, as these attributes are often correlated in real-world settings. Our current approach employs the outer product of independent probabilities as a baseline approximation of the joint distribution. However, if external constraints (such as census data or administrative statistics) are available, an iterative proportional fitting process could be applied to harmonize these estimates by reweighting their values to conform with one another^61^.

Another key limitation is our coarse categorization of use types. The model currently operates at the level of eight broad categories (e.g. ”Residential” instead of ”Rural Multifamily”, ”Retail” instead of ”Coffee Shop”), meaning that some buildings are assigned generalized occupancy rates instead of more context specific ones. As finer-grained training data become available, an improved classification system could incorporate hierarchical category assignments to refine these occupancy rates and further improve population model outputs.

Geographically, our training data are not evenly distributed within many countries, with labeled datasets disproportionately concentrated in urban centers while rural and peri-urban areas remain underrepresented. This spatial bias can introduce systematic errors in population estimates, particularly in regions with little or no labeled data. As a result, population estimates in these underrepresented regions may be less reliable. A key priority for future work is to systematically identify regions sparse training data coverage and incorporate additional labeled data – through targeted annotation efforts or integration of auxiliary datasets – to improve model robustness across different geographic settings.

Another assumption in our model is that building area is completely known and as such is static, which affects our population estimate calculations. In reality, the usable interior space of a structure often diverges from its planar footprint, particularly in complex buildings such as multi-use structures, buildings with large empty spaces, or partially occupied spaces. This discrepancy can introduce bias into population estimates that rely on floor area as a key disaggregation factor. In future work, we will explore approaches to estimate a probability distribution over building area adjustments, incorporating either empirical urban design rules or remote sensing techniques.

The current population estimation model is inherently simple, relying on the product of density rates and occupiable space. Incorporating additional parameters, such as vacancy rates, building condition, and socioeconomic classifications, offers a promising direction for further improving population estimates in LandScan. While these variables are frequently discussed in the literature, ensuring their global availability and scalability pose significant barriers. Future work will focus on adapting existing methodologies or developing new ones that meet LandScan’s worldwide deployment requirements, enabling more accurate and comprehensive population modeling. Despite the inherent simplicity of the population function, one challenge observable in the current methodology is the complexity and computational cost of implementing it, as it scales linearly with the total number of buildings present within a country. As such, applying the workflow will be demanding in densely built countries. Future work will emphasize this challenge and develop surrogate supervised machine learning models, where LSM output across different percentiles can be used as training. These surrogate models would act as approximations, enabling rapid inference of population distributions without incurring full computational cost.

Lastly, because LSM is a building-level population model, any errors in building footprint extraction from imagery will propagate through the population estimation process. Errors in footprint delineation, such as inaccuracies in capturing the true on-ground extent of buildings or misclassifications in presence and absence, introduce distinct challenges for different components of the model. For the use type and floor count models, which rely on building morphology, inaccuracies in footprint shape, representation, or coverage can reduce predictive accuracy, leading to misclassified building types and unreliable floor estimates^28,39^. Additionally, errors in building footprint coverage, such as false positives and false negatives, can distort population distributions. This may lead to unrealistic population estimates in certain geographic areas, particularly when evaluated against in-situ data. Addressing these challenges may require incorporating uncertainty aware footprint extraction methods or integrating multiple data sources to improve footprint reliability. By addressing these limitations, we aim to improve the accuracy, generalizability, and interpretability of population distributions for the high-stake, application-oriented fields that rely on population estimates.

## Methods

In this section, we outline the methodological modifications to the LSHD population modeling workflow. The primary modification to the workflow is a shift to using probability distributions as the primary modeling inputs. As such, in this section we also discuss the model architecture to derive the probability distributions for building level floor count and use type attributes, needed for population estimation. In addition, we provide detail on the methodology of how the PDT building use type specific occupancy rates are derived and provide suggested guidance for how to leverage the uncertainty derived from the LSM empirical distribution.

### Population model

The generalized modeling approach for LSHD involves three main stages: (1) estimating a bottom-up population at the building level, (2) normalizing these estimates to match a known total population, and (3) aggregating to grid cells. Here we detail this and introduce our revised approach that further refines each stage to enable uncertainty quantification and propagation throughout the model.

#### LandScan HD approach

Let *B* be the set of buildings in the country of interest. Each building $b \in B$ is assigned a floor count $F_b$, a use type *u*(*b*), and a country-specific occupancy rate (people per 1000 sqft) corresponding to use type $O_{u(b)}$,. If either use type or floor count is missing, a default constant value is used:

1a $$\begin{aligned} u(b)&= {\left\{ \begin{array}{ll} {\hat{u}}(b), & \text {if known},\\ \textrm{residential}, & \text {otherwise}, \end{array}\right. } \end{aligned}$$

1b $$\begin{aligned} F_b&= {\left\{ \begin{array}{ll} {\hat{F}}_b, & \text {if known},\\ \bar{F}, & \text {otherwise}, \end{array}\right. } \end{aligned}$$

where $\bar{F}$ typically is a value between one and two. Otherwise, conflated labels or known data provide ${\hat{u}}(b)$ and ${\hat{F}}_b$. Once a building is classified with a use type *u*(*b*) and a floor count $F_b$, its corresponding occupancy rates for various time periods $t_i$ are retrieved from use type-specific occupancy beta distributions, using the 50th percentile as the representative value (2).

2 $$\begin{aligned} O_{t_i, u(b)} = \text {Q}_{0.5}\text {Beta}(\alpha _{t_i, u}, \beta _{t_i, u}) \cdot O_{\max , t_i, u}, \quad \scriptstyle {\text {for } i = 1, \dots , n} \end{aligned}$$

where $O_{\max , t_i, u}$ is an upper bound estimate on the occupancy rate for time period $t_i$ among all time periods *n* and use type *u* (see the *Facility Type Occupancy Rates* Section for further details).

This yields preliminary population estimates for building *b* at each time period $t_i$:

3 $$\begin{aligned} {\hat{p}}_{t_i, b} = A_b \cdot F_b \cdot \frac{O_{t_i, u(b)}}{1000}, \quad \text {for } i = 1, \dots , n . \end{aligned}$$

where $A_b$ represents the building footprint area. These time specific estimates are then combined to produce an initial ambient population estimate for building *b*:

4 $$\begin{aligned} {\hat{p}}_b = \frac{\sum _{i=1}^{n} w_i {\hat{p}}_{t_i, b}}{\sum _{i=1}^{n} w_i} \end{aligned}$$

where $w_i$ denotes the weight associated with each time period $t_i$. Next, let *P* be an authoritative total population for the entire region. The model normalizes all ${\hat{p}}_b$ values so that they sum to *P*:

5 $$\begin{aligned} p_b = \frac{{\hat{p}}_b}{\sum _{b' \in B} {\hat{p}}_{b'}} \cdot P, \end{aligned}$$

thus ensuring $$\sum _{b \in B} p_b = P$$.

The target output is a raster composed of 3-arc-second grid cells, indexed by $g$ and denoted $G_g$. Because a single building footprint $F_b$ can intersect several of these predefined cells, assigning the entire value $p_b$ to just one cell would distort the spatial distribution of population. To avoid this distortion, we split the footprint along grid boundaries; every split intersection with cell $G_g$:

$$M_{b,g}=F_b\cap G_g,$$

is treated as a distinct building sub-component, or *molecule*^11,25^.

Each molecule inherits a fraction of its parent building’s population according to the share of footprint area it represents:

$$q_{b,g} \;=\; \frac{\operatorname {area}(M_{b,g})}{\operatorname {area}(F_b)}, \qquad \sum _{g} q_{b,g} \;=\; 1 .$$

The population assigned to molecule (*b*, *g*) is therefore:

6 $$\begin{aligned} p_{b,g} \;=\; q_{b,g}\, p_b. \end{aligned}$$

Finally, the total population in grid cell *g* is obtained by summing over all molecules lying in that cell:

7 $$\begin{aligned} p_g \;=\; \sum _{b \in B} p_{b,g} \;=\; \sum _{b \in B} q_{b,g}\, p_b, \end{aligned}$$

thus concluding the LSHD workflow.

#### Revised LandScan mosaic approach

The LSHD method relies on fixed values for building use type, floor count, and occupancy rate, assigning defaults when labels were absent. In the LSM framework, we adopt a probabilistic approach, leveraging two ML models.

For each building $b$, an ML classifier outputs a probability distribution over possible use types and floor counts:

8a $$\begin{aligned}&P(U_b = u) = \text {ML}_{U}(X_b; \theta _U), \quad u \in {\mathcal {U}}, \end{aligned}$$

8b $$\begin{aligned}&P(F_b = f) = \text {ML}_{F}(X_b; \theta _F), \quad f \in {\mathcal {F}}. \end{aligned}$$

Equations (8a) and  (8b) represent the prediction probabilities of each use type $u$ and building floors $f$. Because these two variables can be interrelated, we do not sample them independently. Instead, we derive a conditional distribution $P(F_b = f \mid U_b = u)$ by combining the probabilities from (8a) and (8b). First, we construct an unnormalized joint distribution via an outer product. Then, we normalize it to obtain a joint probability:

9a $$\begin{aligned} {\tilde{P}}(U_b = u, F_b = f)&= P(U_b = u)\;\cdot \;P(F_b = f), \end{aligned}$$

9b $$\begin{aligned} P(U_b = u,\, F_b = f)&= \frac{ {\tilde{P}}(U_b = u,\, F_b = f) }{ \sum _{u'\!,\,f'} {\tilde{P}}(U_b = u'\!,\,F_b = f') }. \end{aligned}$$

Then, to sample floors conditional on use type, we compute

10 $$\begin{aligned} P(F_b = f \,\mid \, U_b = u) =\; \frac{P(U_b = u,\, F_b = f)}{\sum _{f'} P(U_b = u,\, F_b = f')}. \end{aligned}$$

As such, once we sample $U_b$, the feasible floor counts $F_b$ reflect that choice.

We conduct $K$ Monte Carlo draws per building to produce an empirical distribution of population estimates. On the $k$-th draw, we:

1. Sample a use type:
  11 $$\begin{aligned} u_b^{(k)} \sim P(U_b = u). \end{aligned}$$
2. Sample a floor count given the sampled use type:
  12 $$\begin{aligned} f_b^{(k)} \sim P(F_b = f \mid U_b = u_b^{(k)}). \end{aligned}$$
3. Sample a time-specific occupancy rate for each time period $t_i$:
  13 $$\begin{aligned} o_{t_i, b}^{(k)} \sim {\mathcal {O}}_{t_i, u_b^{(k)}}, \quad \text {for } i = 1, \dots , n, \end{aligned}$$
  where ${\mathcal {O}}_{t_i, u_b^{(k)}}$ is the empirical occupancy distribution for a use type $u_b^{(k)}$ at $t_i$, constructed as described in Eq. (27).
4. Compute time-specific population estimates for draw $k$:
  14 $$\begin{aligned} p_{t_i, b}^{(k)}&= A_b \cdot f_b^{(k)} \cdot \frac{o_{t_i, b}^{(k)}}{1000} \\&\scriptstyle {\text {for } i = 1, \dots , n} \nonumber \end{aligned}$$
5. Compute the ambient population estimate ${p}_b^{(k)}$ for draw $k$ as the weighted average of time-specific estimates using $w_i$:
  15 $$\begin{aligned} {p}_b^{(k)} = \frac{\sum _{i=1}^{n} w_i p_{t_i, b}^{(k)}}{\sum _{i=1}^{n} w_i} \end{aligned}$$

By repeating these steps $K$ times, we generate a distribution of ambient population estimates for each building, incorporating uncertainties in use type, floor count, and occupancy rates across multiple time periods that were absent in the LSHD approach.

After $K$ draws, the mean building-level population estimate is

16 $$\begin{aligned} {\hat{p}}_b = \frac{1}{K} \sum _{k=1}^{K} p_b^{(k)}, \end{aligned}$$

and we record the 5th and 95th percentiles ($p_b^{(0.05)}$ and $p_b^{(0.95)}$) from the sampled values $\{p_b^{(k)}\}_{k=1}^K$ to quantify uncertainty.

Let $P$ be the authoritative total population for the region. We define a single scaling factor $\alpha$ based on the sum of mean estimates:

17 $$\begin{aligned} \alpha = \frac{P}{\sum _{b' \in B} {\hat{p}}_{b'}}, \end{aligned}$$

ensuring that $\sum _{b \in B} \alpha \,{\hat{p}}_b = P$. We apply this same factor $\alpha$ to the 5th and 95th percentile estimates, preserving their relative uncertainty:

18 $$\begin{aligned} p_b^{(q)} = \alpha \,{\hat{p}}_b^{(q)}, \quad q \in \{0.05, 0.50, 0.95\}. \end{aligned}$$

Using the molecule?based proportions $q_{b,g}$ introduced in Eq. (6), each building?level quantile is first apportioned to its intersecting grid cells:

19 $$\begin{aligned} p_{b,g}^{(q)} \;=\; q_{b,g}\, p_b^{(q)}, \qquad q \in \{0.05, 0.50, 0.95\}. \end{aligned}$$

The quantile value for grid cell $g$ is then the sum over all molecules that fall in that cell:

20 $$\begin{aligned} p_g^{(q)} \;=\; \sum _{b \in B} p_{b,g}^{(q)} \;=\; \sum _{b \in B} q_{b,g}\, p_b^{(q)}, \qquad q \in \{0.05, 0.50, 0.95\}. \end{aligned}$$

This mirrors the final step of the LSHD approach but now accounts for the range of plausible values at the building level, aggregated to the grid cell. As a result, the grid-cell population estimates include both a mean value and a prediction interval.

### Building use type model

To estimate the probability distribution of building-level use types $U_b$, we adapt a tree-based classification methodology following Adams et al.^33,38,39^, originally used to distinguish residential vs. non-residential structures. Rather than a binary distinction, however, we assign each building $b$ a probability over eight broad use type categories: *Residential, Institutional, Retail, Commercial, Transportation, Military, Recreation,* and *Agriculture*.

#### Classification approach

Let $\{{\mathcal {T}}_t\}_{t=1}^T$ be the $T$ trees in our random forest classifier, each voting for one of the eight categories. We aggregate votes across all trees to derive a categorical probability distribution:

21 $$\begin{aligned} P\bigl (U_b = u\bigr ) \;=\; \frac{1}{T} \sum _{t=1}^T \textbf{1}\bigl ({\mathcal {T}}_t(b) = u\bigr ), \quad u \in {\mathcal {U}}. \end{aligned}$$

This enables the model to express uncertainty regarding the building’s use type based on the ambiguity present in its morphological profile.

#### Data and features

As shown in Table 4, we employ morphological features to train our random forest model. Labeled training data are obtained primarily from OSM^29^, and augmented by the Urban Tactical Planner (UTP)^62^ and Multinational Geospatial Co-production Program (MGCP). Conflation with the building footprints follows the approach of Tuccillo et al.^11^ and Moehl et al.^25^. When multiple labels are present for a single building, we assign the largest proportional label, thus creating a single “dominant” class for supervised learning.

#### Probabilistic output

Upon inference, each tree in the forest produces a vote. Aggregating these votes yields a probability distribution over the eight categories, indicating the model’s confidence in each. For instance, if half the trees vote “Residential” and the rest are split among “Commercial” and “Retail,” the resulting probability vector might read $\{ \textrm{Res}: 0.5,\; \textrm{Com}: 0.3,\; \textrm{Ret}: 0.2 \}$. In the downstream Monte Carlo simulation, these probabilities guide which use type $u_b$ is sampled for each building, providing a realistic reflection of uncertainty about the building’s function, based on its morphological profile.

### Building floor count model

To estimate the probability distribution for the number of floors $F_b$, we adapt tree-based regression methodologies shown in Stipek et al.^28^ and Adams et al.^39^ initially designed to predict raw building height. Rather than training a random forest classifier directly, we use a *regressor* and then transform its continuous predictions into a discrete distribution. We model floors in this manner to address a common limitation in available floor count label data, where examples for all possible floor count classes are often missing. This constraint makes framing the problem as a classification task, akin to the use type likelihood model, impractical. We instead treat floor counts as a continuous variable, allowing the model to predict values not present within the training set of values. We then discretize these continuous predictions to the nearest floor count classes to obtain tree-specific predictions. This approach allows us to aggregate tree predictions in a manner similar to a traditional random forest classifier, thereby deriving our floor count class-specific probabilities.

#### Regression and rounding

Let $\{{\mathcal {T}}_t\}_{t=1}^T$ be the $T$ trees in our random forest regressor, each producing a continuous floor estimate $f^t(b)$ for building $b$. We *round* these estimates:

22a $$\begin{aligned} {\hat{F}}_b^t&= \textrm{round}\bigl (f^t(b)\bigr ), \end{aligned}$$

22b $$\begin{aligned} P(F_b = f)&= \frac{1}{T} \sum _{t=1}^T \textbf{1}\bigl ({\hat{F}}_b^t = f\bigr ), \quad f \in {\mathcal {F}}, \end{aligned}$$

so a predicted value of $2.09$ becomes $2$, while $1.1$ becomes $1$, and so on. This converts the regressor’s continuous outputs into a discrete probability distribution over possible floor counts $f \in {\mathcal {F}}$.

#### Data and features

We train the random forest regressor using the same feature set (see Table 4) as the use type likelihood model and derive labels from the same sources. Additionally, the conflation process follows the same methodology as in the use type model.

#### Probabilistic output

From Eq. (22b), we obtain a full probability vector over ${\mathcal {F}}$. For instance, if half the trees predict $1$ floor and the rest predict $2$ floors, then $P(F_b = 1) = 0.5$ and $P(F_b = 2) = 0.5$. In the downstream Monte Carlo simulation, these probabilities guide which floor count $f_b$ is sampled for each building, providing a realistic reflection of uncertainty about the building’s function.

**Table 4:** Building morphology features used for the use type and floor count models.

Indicator	Description
Geometric properties	
sqft	Area in square feet
shape_length	Perimeter length
envel_area	Area of bounding box of geometry
vertex_count	Count of vertices in the geometry
geom_count	Count of polygons in the geometry
complexity_ratio	Perimeter/Area, measure of shape complexity
iasl	Inverse average segment length
vpa	Vertices per area
complexity_ps	Complexity per segment, average complexity within each segment
ipq	Isoperimetric quotient, shape area maximization for given perimeter length
Spatial properties	
lat_dif	Max latitude minus min latitude
long_dif	Max longitude minus min longitude
nnd	Nearest Neighbor Distance, measured between centroids
n_count (50, 100, 250, 500, 1000)	Number of building centroids within buffer ranges
omd (50, 100, 250, 500, 1000)	Observed mean distance within buffer ranges
emd (50, 100, 250, 500, 1000)	Expected mean distance for uniform spacing
nni (50, 100, 250, 500, 1000)	Nearest Neighbor Index within buffer ranges
Intensity (50, 100, 250, 500, 1000)	Nearest Neighbor Index within buffer ranges
Contextual properties	
n_size_mean (50, 100, 250, 500, 1000)	Average building size within buffer ranges (sqft)
n_size_std (50, 100, 250, 500, 1000)	Std. dev. of sizes within buffer ranges (sqft)
n_size_min (50, 100, 250, 500, 1000)	Minimum building size within buffer (sqft)
n_size_max (50, 100, 250, 500, 1000)	Maximum building size within buffer (sqft)
n_size_cv (50, 100, 250, 500, 1000)	Coefficient of variation of sizes within buffer (sqft)

### Facility type occupancy rates

There are two objectives in modeling occupancy ($\text {people}/1000\text {ft}^2$) within each facility type. The first is to capture natural variations in ambient occupancy that occur across buildings. These variations arise from latent processes such as venue popularity, use schedules, and local patterns of life. Natural variation is modeled as a Beta distribution over $(0, O_{\max })$, where $O_{\max }$ is a practical upper bound on the number of humans that can reasonably fit within a $1000\text {ft}^2$ space.

The Beta distribution, shown in Eq. (23), is highly flexible and can represent a wide range of shapes, from highly skewed to approximately Gaussian or even relatively uniform:

23 $$\begin{aligned} pdt_{bt}\sim \textrm{Beta}(\alpha ,\beta ). \end{aligned}$$

where $pdt_{bt}$ , is the model characterizing ambient occupancy for buildings within a specified taxonomy (e.g. museums).

The second objective is to capture uncertainty about these natural variations through uncertainty in the parameters of the Beta distribution. Parameter uncertainty is driven both by a frequent lack of data and the uncertainty in that data. In practice, building specific ambient occupancy (the data) is not actually observable but must be inferred from other observable properties such as area, number of floors, employees, visitors, and hours of operation, as in Eq. (24). Since these observables are themselves typically uncertain, we model them as random variables (e.g. uniform, Gaussian) opportunistically designed to capture a wide range of approaches for reporting uncertainty found in the open source. From these, we develop probabilistic observation models, such as the museum model adapted from Stewart et al.^14^, below:

24 $$\begin{aligned} p(\textrm{pdt} \mid \omega ) \sim 1000 \cdot A^{-1} \left[ \frac{a \, \cdot \overline{v}}{{d}\, \cdot \overline{h}} + w \, \cdot \overline{p} \right] \end{aligned}$$

where $p(\text {pdt} \mid \omega )$ is an observation model of museum ambient occupancy (in $\text {people}/1000\,\text {ft}^2$), and $\omega$ is a vector of values that includes:

- $a$: annual number of visitors,
- $\overline{v}$: average visit time,
- $d$: number of days open,
- $\overline{h}$: average number of daily hours,
- $w$: number of employees,
- $\overline{p}$: average percent of employees working at any given time,
- $A$: museum floor area.

Morton^63^ demonstrates via proof that such observation models and their inputs constitute necessary and sufficient conditions to estimate latent ambient occupancy. For more information on the observation models currently in use by PDT, see Stewart et al.^14^ and Urban et al.^12^.

Because the number of observational data points is typically low, we adopt a Bayesian approach in which subject matter expertise, aggregated summary data sets, and experiential knowledge can be encoded as priors on the model parameters, $p(\alpha , \beta )$. The approach to encoding and constructing these priors will depend on the circumstances, however Stewart et al.^64^ articulates a generic approach by which elicited knowledge may be encoded as a bivariate Gaussian prior. The parameter posterior distribution is addressed using Jeffrey’s rule of probability kinematics^65^, which provides a conflation over probabilistic observation models:

25 $$\begin{aligned} p(\alpha , \beta \mid \omega ) \;=\; \int p(\alpha , \beta \mid pdt)\; p(pdt \mid \omega ) \; dpdt, \end{aligned}$$

26 $$\begin{aligned} p(\alpha , \beta \mid pdt) \;=\; \frac{p(pdt \mid \alpha , \beta )\, p(\alpha , \beta )}{\displaystyle \int p(pdt \mid \alpha , \beta )\, p(\alpha , \beta )\, d_{\alpha \beta }}. \end{aligned}$$

The final posterior can be sampled over $(\alpha , \beta )$ to assess uncertainty in the range of possible Beta distributions. A single parameter estimate can be derived over posterior samples by a weighted average $\bigl (\overline{\alpha }, \overline{\beta }\bigr )$, ensemble modeling, or other conflation approaches.

#### Broad category occupancy rates

PDT reports 65 facility types^26^, each described by shape parameters $\alpha _v, \beta _v$ and an upper limit $O_{\max ,v}$, where $v$ represents a specific facility type. However, many of the building attribution sources used by LSHD report buildings at a higher-level category (e.g. ‘retail structure’ instead of ‘coffee shop,’ or ‘residential structure’ instead of ‘single-family house’).

To accommodate these broader attributions, we group the 65 facility types into 8 broad use type categories ${\mathcal {U}}$ (i.e. *Residential, Institutional, Retail, Commercial, Transportation, Military, Recreation,* and *Agriculture*). The variable $U_b$, used in the classification model Eq. (8a), maps each building to one of these categories and serves as the linking field for assigning occupancy information. For each broad use type $u \in {\mathcal {U}}$, we construct an empirical occupancy distribution by drawing 10,000 samples from each detailed facility type $v \in u$, using the Beta distribution with parameters $(\alpha _v, \beta _v)$ scaled by the corresponding upper bound $O_{\max ,v}$, and aggregating the results as:

27 $$\begin{aligned} {\mathcal {O}}_u = \bigcup _{v \in u} \left\{ o_v^{(j)} \sim \text {Beta}(\alpha _v, \beta _v) \cdot O_{\max ,v} \right\} _{j=1}^{N} \end{aligned}$$

The resulting set ${\mathcal {O}}_u$ defines the empirical distribution for the broad use category. The maximum of the contributing $O_{\max ,v}$ values is retained as the category-level upper limit $O_{\max ,u}$. This approach effectively replaces the LSHD practice which relied on averaging the medians of subcategory occupancy rates to create $O_{u(b)}$ at $t_i$ in Eq. (2) to accommodate instances of broad category building labels.

### Uncertainty-weighted adjustments to population estimates

The LSM framework produces an empirical population distribution for each building modeled, through repeated sampling of model uncertainty. Each draw incorporates variability from three key components: the predicted class probabilities for building use type, the predicted floor count distribution, and the occupancy rate derived from the PDT model’s Bayesian posterior. The median $P_{50}$ of this empirical distribution provides a robust central estimate that can be calbirated to authoritiative census totals, while the associated $P_{5}$ and $P_{95}$ percentiles preserve information about relative confidence and potential asymmetry in the modeled outcomes (see Eqs. 17 and 18).

While these percentiles effectively communicate unceratinty, they do not directly inform how such uncertainty should influence decision-making. In many applications, be it exposure assessment or resource prioritization, decision-makers may wish to explicitly control the influence of confidence or to emphasize either the risks of overcounting or undercounting. To support this perceived need, we introduce a uncertainty-weight adjustment applied to the median population estimate:

28 $$\begin{aligned} {\tilde{P}} = F \, \cdot \, P_{50}, \end{aligned}$$

where the adjustment factor *F* is defined as

29 $$\begin{aligned} F = 1 + \gamma \big ( \omega _{u}\, \phi - \omega _{d}\, \delta \big ) + (1 - \gamma )\, \omega _{\sigma }\, \mu \, \eta . \end{aligned}$$

where,

- $\gamma$: confidence coefficient derived from the normalized spread between the 95th and 5th percentiles:
  $$\gamma = 1 - \frac{P_{95} - P_{5}}{P_{95} + P_{5}}.$$
- $\phi$: upside fractional deviation (indicative of a potential undercount):
  $$\phi = \frac{P_{95} - P_{50}}{P_{50}}.$$
- $\delta$: downside fractional deviation (indicative of a potential overcount):
  $$\delta = \frac{P_{50} - P_{5}}{P_{50}}.$$
- $\mu$: magnitude of uncertainty, representing the total fractional spread of the empirical distribution:
  $$\mu = \phi + \delta .$$
- $\eta$: normalized directional skew, indicating the relative dominance of undercounting or overcounting risk:
  $$\eta = \frac{\phi - \delta }{\mu + \varepsilon }, \qquad \varepsilon > 0.$$
- $\omega _{u},\, \omega _{d}$: directional weighting coefficients governing the relative influence of upside (undercounting) versus downside (overcounting) risk.
- $\omega _{\sigma }$: global weighting coefficient controlling the overall influence of uncertainty magnitude on the adjustment.
- $\varepsilon$: small positive constant used to prevent division by zero when $\mu = 0$.

This formulation ensures that the adjusted population ${\tilde{P}}$ shifts in proportion to both the confidence and directional asymmetry of the empirical distribution. When uncertainty is low or symmetric ($U \approx D$, $C \approx 1$), the factor approaches unity ($F \approx 1$), yielding ${\tilde{P}} \approx P_{50}$. In contrast, when uncertainty is high and skewed, *F* diverges from unity, amplifying or muting the estimate to reflect the modeled directional risk.

### Demonstration

To illustrate the practical impact of replacing deterministic assumptions with a ML driven framework that incorporates uncertainty quantification and propagation, we replicate the spatial analysis using LSHD estimates from Tuccillo et al.^11^, which estimates populations susceptible to flooding events. Expanding upon the LSHD approach, we implement a SDM framework to rank administrative units within the city based on projected flood-impacted population counts. In addition to the LSHD dataset, we evaluate two outputs from our probabilistic LSM framework: the median estimate and an uncertainty adjusted variant (LSM-UA). We also include the WP dataset for comparative grounding.

By configuring the demonstration as a multi-dataset comparison, we emphasize the sensitivity of structured decision-making processes to the incorporation of ML-driven predictive modeling and uncertainty quantification in population data. This analysis underscores the critical role that ML-enhanced, probabilistic population modeling plays not only in environmental risk assessment but also across a broad range of domains where population data informs evidence-based decision-making and risk management strategies.

#### Background

For this demonstration, we adopt the same study area as in a previous demonstration of the LSHD model: Iloilo City, Philippines^11^. Iloilo City is the provincial capital of Iloilo province and part of a region of the Philippines that is historically vulnerable to recurrent flooding and storm surge events^66,67^. Flood susceptibility data, sourced from the Philippines government’s geospatial data portal^68^, encompass nearly the entire municipal area. To facilitate regional analysis and supported structured decision-making based on estimates of impacted population, we use administrative-level 4 boundaries within the municipality, sourced from the Philippines Statistics Authority.

#### Data

**Fig. 7:**
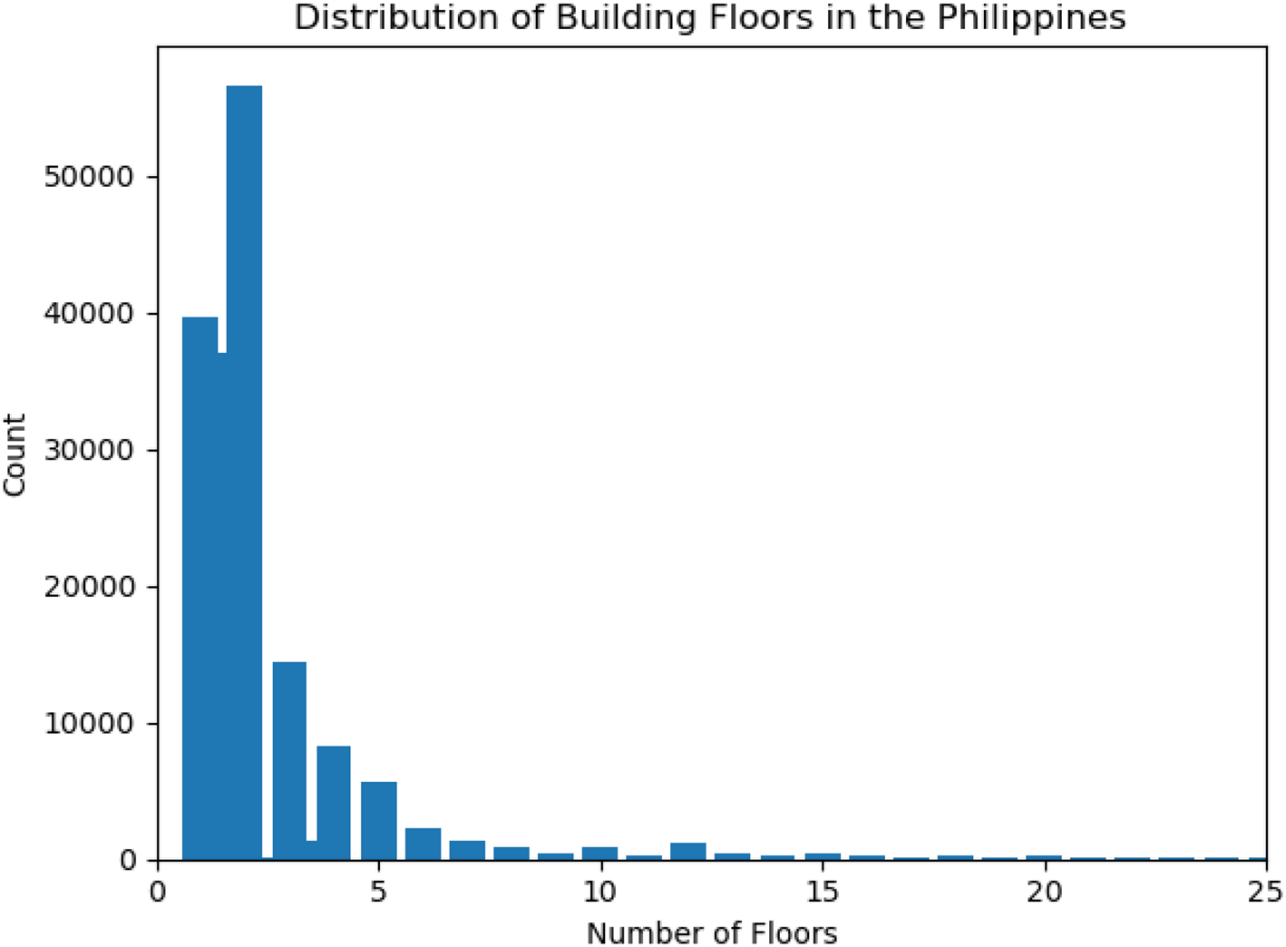
Distribution of predicted floor counts for buildings in the Philippines.

Building footprint data are derived from Microsoft^69^ and Google^70^. Where building footprint coverage are lacking, we fill in the spatial gaps with built up surface information from GHSL, as described in Tuccillo et al.^11^. Table 5 provides an exact count of buildings by their dataset source. Table 6 provides details on the total number of labels for each of our building use type categories. Figure 7 shows the distribution of our labeled floor count data used for the demonstration. To normalize building-level population estimates, we leverage LSG administrative population counts as our authoritative county-level population estimates. These estimates are obtained their values from the United States Census International Database^7,8,22^.

**Table 5:** Total number of buildings by data source used in the Philippines.

Source	Count
microsoft_01302024	29
microsoft_04252023	12,431,598
ghsl_v2023	5,243
google_v3	12,697,710

#### Model setup

We train Random Forest models independently, using a Random Forest Classifier for predicting building use type (8a) and a Random Forest Regressor for estimating floor counts (8b) across the full country-level geography. To optimize model performance we conduct hyper-parameter tuning with *GridSearchCV* from the *scikit-learn* library^71^. We evaluate 80 different combinations from the following integer ranges:

- *max_depth*: {10, 20, 30, 40, Indefinite}
- *min_samples_split*: {2, 5, 10, 20}
- *min_samples_leaf*: {1, 2, 5, 10}

**Table 6:** Labeled building use type counts in the Philippines.

Use type	Count
Residential	18,970,603
Institutional	1,693,986
Retail	215,298
Commercial	1,011,087
Transportation	78,770
Military	27,006
Recreation	87,757
Agriculture	32,351

The grid search evaluates combinations of maximum tree depth, minimum samples required to split a node, and minimum samples required for a leaf node. The evaluation was performed using 3-fold cross-validation, which was selected to balance the trade off between comprehensive model assessment and the computational resources required. The selected setting is based on the best performing configuration, using the macro average F-1 score across all folds. The results for the *GridSearchCV* can be seen in Table 7, while all other hyperparameters were left to their default settings as set by the scikit-learn library^71^, except for the *random_state* which was set to 42 for repeatability.

**Table 7:** Selected hyperparameters for floors regression and use type classification models in the Philippines.

Parameter	Floors model	Use type model
max_depth	30	Indefinite
min_samples_leaf	5	5
min_samples_split	2	20

#### Uncertainty-weighted adjustments configuration

To simulate a SDM analysis from the perspective of a decision-maker responsible for resource allocation and flood mitigation, we configured the LSM-UA to emphasize potential undercounting risk over overcounting risk. This configuration reflects a conservative decision-making stance in which underestimation of exposed population is considered more consequential than that of overestimation. We did this by assigning weighting coefficients of 0.20 for overcounting risk, 0.30 for undercounting risk, and 0.40 for general uncertainty influence. The adjustment factor was constrained to the range of 0.2-2.5 to prevent excessive deflation or inflation of the adjusted population values. This configuration produced an uncertainty-weighted population surface that prioritizes conservative interpretation of a potential exposure while maintaining numerical stability and comparability with the median LSM layer.

#### Preprocessing steps

Prior to configuring the SDM analysis, all population datasets were spatially aligned. Both LSHD and LSM were derived from the same authoritative census totals for a common reference year. To maintain comparability, the WP dataset was normalized at the national scale so that its total population matched the census total used for LSM and LSHD. This normalized controlled for differences in external adjustment sources while preserving the original local spatial distribution patterns of WP for subsequent SDM analysis.

#### Methods section

Here we detail the methodology used to demonstrate how the choice of population dataset influences potential prioritizations in SDM analysis. We extend the comparative framework of Tuccillo et al.^11^ by including four population datasets: LSHD, LSM, WP, and LSM-UA.

Let $p_i^{(d)}$ denote the population value at raster cell *i* for dataset $d \in \{\text {LSHD}, \text {LSM}, \text {WP}, \text {LSM-UA}\}$. The study area is categorized into three flood risk zones:

$${\mathcal {R}} = \{r_{\text {low}},\, r_{\text {moderate}},\, r_{\text {high}}\},$$

numerically encoded as $\{0.33,\, 0.66,\, 1.00\}$ for subsequent computations.

The total population for dataset *d* across the study area is

$$P_{\text {total}}^{(d)} = \sum _{i \in B} p_i^{(d)},$$

where *B* is the set of all raster cells within the analysis boundary. For each flood-risk zone $r_s \in {\mathcal {R}}$, let $A_s$ denote the subset of raster cells assigned to that risk class. The total and proportional population exposed in class *s* are then:

$$P_s^{(d)} = \sum _{i \in A_s} p_i^{(d)}, \qquad \text {pct}_s^{(d)} = \frac{P_s^{(d)}}{P_{\text {total}}^{(d)}} \times 100\%.$$

To enable a spatially explicit SDM comparison, we model exposure as the product of two continuous rasters: population exposure and flood risk. For each cell *i*, the SDM composite score is defined as:

$$S_i^{(d)} = p_i^{(d)} \, r_i,$$

where $r_i$ denotes the normalized flood risk value at cell *i*. This product yields an SDM raster $\textbf{S}^{(d)}$ representing the combined influence of hazard intensity and exposed population for each dataset.

To facilitate comparison across spatial decision-making units, we compute the mean SDM value for each unit $u_j \in U$, where *U* denotes the set of administrative level-4 units in the study area. The mean SDM score for unit $u_j$ and dataset *d* is defined as

$$\bar{S}_j^{(d)} = \frac{1}{|u_j|} \sum _{i \in u_j} S_i^{(d)},$$

where $|u_j|$ represents the number of raster cells contained within unit $u_j$. This aggregation produces a single exposure score per administrative unit, enabling standardized comparison across population datasets.

Each administrative unit is then ranked in descending order according to its mean SDM score, with rank $R_j^{(d)}$ assigned such that the unit with the highest score receives rank 1:

$$R_j^{(d)} = \text {rank}\big (\bar{S}_j^{(d)}\big ), \qquad j = 1,\ldots , |U|.$$

To assess the degree of agreement between prioritization outcomes derived from different population datasets, we compute Spearman’s Rank Correlation Coefficient ($\rho$) for each pairwise comparison of rankings:

30 $$\begin{aligned} \rho = 1 - \frac{6 \sum _{j=1}^{n} d_j^2}{n(n^2 - 1)}, \end{aligned}$$

where:

- $d_j$ is the rank difference between dataset rankings for unit $u_j$,
- $n = |U|$ is the total number of administrative units.

We interpret Spearman’s rank correlation coefficient $\rho$ as follows:

- *High correlation* ($\rho > 0.75$): Rankings are highly consistent across datasets, indicating broad agreement in which administrative units are prioritized for intervention at the spatial scale evaluated.
- *Moderate correlation* ($0.5 < \rho \le 0.75$): Rankings are partially consistent, suggesting that differences in population modeling approaches or uncertainty representation introduce meaningful, but not transformative, adjustments to priority ordering.
- *Low correlation* ($0.25 < \rho \le 0.5$): Rankings diverge substantially, indicating that dataset choice can alter which administrative units are elevated for priority consideration, potentially reshaping decision-making outcomes.
- *Near zero* ($\rho \le 0.25$): Rankings are largely inconsistent, demonstrating that different population models yield fundamentally different prioritization patterns, with potential reversal of which regions are considered highest risk or highest need.

All zonal statistics are calculated using ArcGIS Pro version 3.3.0. While $\rho$ was computed using the scipy library scipy.

## Data Availability

The resulting population and confidence rasters are available upon request of the corresponding author: Daniel Adams, at adamsds@ornl.gov. The datasets will be made available globally at: https://landscan.ornl.gov/ . Building data used in this paper are sourced from commercial and public products. Most building attribution data are obtained via open-source repositories. Code for the recreation of these results and to produce similar results elsewhere using the methodology available upon request.
